# SHAPING, AND BEING SHAPED BY, POLICIES AND PROGRAMS THAT PROMOTE AN AGE-INCLUSIVE WORKFORCE: A PERSONAL JOURNEY

**DOI:** 10.1093/geroni/igae098.0412

**Published:** 2024-12-31

**Authors:** James Westcott

**Affiliations:** University of Colorado Anschutz Medical Campus, Aurora, Colorado, United States

## Abstract

In this session, I will share my personal journey as someone who has both shaped and been shaped by the innovative age-inclusive workforce policies and programming in Colorado. I will describe my state-wide leadership role as part of the Colorado Older Worker Policy Collaborative successfully advocating for the elimination of graduation dates and other age-identifying sections of job applications, which disadvantaged older adults in the job application process. I will also share my experience as an Older Adult Research Specialist who completed a novel pathway to encore careers in research program at the University of Colorado Anschutz Medical Campus. I will share how I apply my dual training as a health navigator and research navigator to recruit and retain older Veterans in clinical trials for the VA Eastern Colorado Geriatric Research Education and Clinical Center – one of 20 centers of excellence focused on aging in the Department of Veterans Affairs. I will describe the meaning of this work to me personally and professionally, as a culmination of many work and life experiences. I will also share the difference I make as an older adult peer for the Veterans with whom I engage in research and on a multi-disciplinary and intergenerational team comprised of an early career investigator and research assistant. My personal and professional journey highlights the importance of a multi-pronged approach, encompassing research, policy and action/application (e.g., education/training and hiring) to promoting the longevity dividend through meaningful work and gainful employment.

